# 

**Published:** 1908-10

**Authors:** 


### BOOKS RECEIVED.

Diseases of the Skin and the Eruptive Fevers. By Jay Frank Schamberg, M.D., Professor of Dermatology and Infectious Eruptive Diseases in the Philadelphia Polyclinic and College for Graduates in Medicine. Octavo of 534 pages, illustrated. Philadelphia and London: W. B. Saunders Company, 1908. (Cloth, $3.00 net.)

---

Gynecology and Abdominal Surgery. In two volumes. Edited by Howard A. Kelly, M.D., Professor of Gynecologic Surgery at Johns Hopkins University; and Charles P. Noble, M.D., Clinical Professor of Gynecology at the Woman's Medical College, Phiadelphia. Large octavo volume of 862 pages, with 475 original illustrations by Mr. Hermann Becker and Mr. Max Brodel. Philadelphia and London: W. B. Saunders Company, 1908. (Per volume: Cloth, $8.00; half morocco, $9.50 net prices.)

---

Anatomy, Descriptive and Surgical. By Henry Gray, F.R.S., late lecturer on Anatomy at St. George's Hospital, London. New American edition, enlarged and thoroughly revised, by J. Chalmers Da Costa, M.D., Professor of Surgery and Clinical Surgery, and Edward Anthony Spitzka, M.D., Professor of Anatomy, in the Jefferson Medical College of Philadelphia. Imperial octavo, 1,625 pages, with 1,149 large and elagorate engravings. Lea & Febiger, Publishers, Philadelphia and New York, 1908. (Cloth, $6.00; leather, $7.00, net prices.)

---

Progressive Medicine, Vol. III, September, 1908. A Quarterly Digest of Advances, Discoveries and Improvements in the Medical and Surgical Sciences. Edited by Hobart Amory Hare, M.D., Professor of Therapeutics and Materia Medica in the Jefferson Medical College of Philadelphia. Octavo, 285 pages, with 30 engravings. Lea & Febiger, Publishers, Philadelphia and New York. (Per annum, in four cloth-bound volumes, $9.00; in paper binding, $6.00, carriage paid to any address.)

---

A Textbook of Physiology. For Students and Practitioners. By George V. N. Dearborn, A.M., (Harvard), Ph.D., M.D. (Columbia), Professor of Physiology in Tufts College, Medical and Dental Schools, Boston. Octavo, 550 pages, with 300 engravings and 8 colored plates. Lea & Febiger, Publishers, Philadelphia and New York, 1908. (Cloth, $3,75 net.)

General Surgery. By Ehrich Lexer, M.D., Professor of Surgery in the University of Koenigsberg. American edition edited by Arthur Dean Bevan, M.D., Professor and head of the Department of Surgery, Rush Medical College, (University of Chicago). An authorised translation of the second German edition by Dean Lewis, M.D., Assistant Professor of Surgery, Rush Medical College. Octavo, pp. 1041. With 449 illustrations and two colored plates. New York and London: D. Appleton & Co.

Fourth Annual Report of the Henry Phipps Institute for the study, treatment and prevention of tuberculosis. February 1, 1906 to February 1, 1907. Edited by Joseph Walsh, A.M., M.D.

Transactions of the thirty-fourth annual meeting of the Oregon State Medical Association held at Portland, Oregon, July 1, to 3, 1908. William House, M.D., secretary.

The Cure of Rupture by Paraffin Injections by Charles C. Miller, M.D. Small 12 mo, pp. 81. Published by the Author, 70 State St., Chicago. (Prepaid $1.00.)

